# Dream Experiences During Intensive Care Unit Stay: Occurrence, Content, Vividness and Associated Factors

**DOI:** 10.1111/nicc.70106

**Published:** 2025-07-02

**Authors:** Adrienne E. van der Hoeven, Rolf Fronczek, Denise Bijlenga, Sarah Hadra, Caro Ridder, Marlies Henneman, Jessica A. Maas, Suzanna A. Goedemans‐de Graaf, Gert Jan Lammers, David J. van Westerloo, Mink S. Schinkelshoek

**Affiliations:** ^1^ Department of Neurology Leiden University Medical Center Leiden the Netherlands; ^2^ Stichting Epilepsie Instellingen Nederland Sleep‐Wake Center Heemstede the Netherlands; ^3^ Department of Intensive Care Leiden University Medical Center Leiden the Netherlands; ^4^ Aftercare Working Group, Department of Intensive Care Leiden University Medical Center Leiden the Netherlands

**Keywords:** critical care, dreaming, intensive care unit, vivid dream experiences

## Abstract

**Background:**

Vivid dream experiences in the intensive care unit (ICU) are common, but poorly understood.

**Aim:**

We investigated the occurrence, vividness, content, emotional impact and associated factors of dream experiences in the ICU.

**Study Design:**

Retrospective mixed methods study with subjects ≥ 18 years, previously admitted to the ICU for ≥ 4 days and/or due to COVID‐19, who were not sedated for ≥ 24 h during their stay (*n* = 80). Participants answered a retrospective questionnaire by telephone. Clinical data were collected from electronic health records.

**Results:**

The questionnaires were taken a median of 9 months post‐discharge. Dream experiences were reported by 79%. Of participants who recollected dream experiences, 73% reported “life‐like” dreams, 49% associated their dreams with negative emotions and 54% said their dreams impacted them even after awaking. Some participants (28.6%) continued to have similar dreams at home. After being asked if they had additional comments, some participants suggest receiving information during their hospital stay about the potential for vivid dream experiences could be beneficial. The dream content was often related to the ICU admission. Younger age and longer length of stay were related to vivid dream experiences. Of participants with dream experiences 62.5% had experienced delirium during their ICU stay. Perceptual disturbances were also frequently reported by participants (50%) and only 45% could clearly distinguish them from dream experiences. There was an overlap between participants reporting perceptual disturbances and confirmed delirium (70%).

**Conclusions:**

Vivid dream experiences are common in ICU patients and often have a negative emotional impact. Future studies should dive deeper into effective ways to distinguish dreams, delirium and perceptual disturbances and how to reduce their impact.

**Relevance to Clinical Practice:**

ICU nurses should be aware of the occurrence and psychological burden of vivid negative dreams in ICU patients. Providing anticipatory support may help patients process these experiences during recovery.

**Summary**

- What is known about the topic
  ○ Sleep difficulties are common in the ICU.
  ○ Literature suggests that vivid and unsettling dreams are frequent among ICU patients, but the number of studies, sample sizes and generalizability have been limited.
  ○ Dream experiences, perceptual disturbances and delirium have been conflated in previous literature.
- What this paper adds
  ○ Vivid dream experiences during ICU admission were reported by 57.5%.
  ○ Dreams were often related to the ICU stay, characterized by negative emotions and impacted patients even after awakening.
  ○ There are substantial overlaps between dream experiences, delirium and perceptual disturbances, with many patients unable to distinguish these phenomena.
  ○ This study increases awareness about dream experiences in the ICU and underscores the need for ongoing psychological support and monitoring during and after ICU care.

## 1 Introduction

In critically ill patients, ensuring restorative sleep is important, as disrupted sleep is associated with impaired cognition, delirium, immune dysfunction and neuroendocrine stress system changes [1]. Sleep disruption and alterations of the circadian rhythm are prevalent in the intensive care unit (ICU) due to a variety of factors, including patient care activities, severity of illness, pain, discomfort, stress, medications and mechanical ventilation [2]. Despite substantial research on sleep in the ICU [2, 3], the investigation into dream experiences in this setting remains insufficient.

## 2 Background

This study was initiated by an internal report from nurses providing ICU aftercare, revealing that many former ICU patients described experiencing strange, vivid and often negative dreams. Our aim was to determine the prevalence of such experiences in the ICU and to identify associated factors.

Literature suggests that vivid and often unsettling dreams are frequent in critically ill patients [4, 5, 6], causing stress even after leaving the ICU [7]. Factors such as length of stay (LOS), mechanical ventilation, conditions such as delirium or Guillain‐Barré Syndrome, and certain medications are thought to be associated with these dream experiences [4, 8, 9, 10, 11, 12].

Previous studies have shown inconsistencies in the frequency and content of dreams and hallucinations, and often conflate the two phenomena [4, 8, 12, 13]. Some literature suggested a possible overlap or shared aetiology between them [14, 15]. However, Cochen et al. (2005) make a clear distinction, defining brief perceptions occurring without any external stimulus, in a state of clear consciousness, whereas vivid dreams are described as prolonged, detailed experiences with a complex narrative that only occur during sleep [6]. In that study, only a small proportion of ICU patients reported vivid dreams and hallucinations, but the overlap between these experiences and their relationship to ICU stay duration was not reported, posing a considerable limitation. Other studies report much higher prevalence rates [4, 8], emphasizing the need for further research to clarify the scope of the issue.

These inconsistencies may be partly due to differing interview times post‐hospitalization and differences in inclusion criteria, particularly concerning the duration of ICU admission. Additionally, existing studies tend to focus on certain patient groups within the ICU, such as those that were delirious, mechanically ventilated or sedated [6, 13]. Lastly, the sample sizes of these studies were relatively small [4, 5, 7, 8, 9, 16, 17, 18, 19, 20]. Larger data sets and broader inclusion criteria could provide a more comprehensive understanding of dream experiences in the overall ICU population.

## 3 Aim and Objectives of Study

We used broad inclusion criteria in a general ICU population to assess the (1) extent, (2) content, (3) impact and (4) related factors of dream experiences in the ICU in general, and vivid dream experiences in particular. We also explored the (5) overlap between delirium, dream experiences and perceptual disturbances. The findings of this study can provide further insights for the improvement of care provided to ICU patients during and after their hospitalization.

## 4 Design and Methods

This study employed a retrospective mixed methods approach, combining quantitative analysis with descriptive accounts, involving former ICU patients who completed a questionnaire via telephone. The study was reported in accordance with the STROBE (Strengthening the Reporting of Observational Studies in Epidemiology) guidelines for observational studies.

### 4.1 Setting

The former patients had been admitted to a mixed ICU at a large tertiary public metropolitan hospital in the Netherlands.

### 4.2 Participants

From June 2021 to November 2022, we reached out to former ICU patients. Inclusion criteria mirrored those guiding ICU aftercare participation (a standard practice for this ICU where former patients with a long admission duration are called 3–4 months after being discharged to see how they are doing) and consisted of:

- Age ≥ 18 years.
- LOS in the ICU of ≥ 4 days and/or admittance due to COVID‐19. Patients admitted for COVID‐19 were included regardless of their ICU stay, consistent with our ICU's aftercare practices and in recognition of the unique nature of this subgroup.
- Not sedated (as measured using a Richmond Agitation‐Sedation Scale score of −1 or higher) for at least 24 h during the ICU admission period.
- The ability to provide informed consent and answer questions via telephone.
- Discharged from the ICU less than a year ago.

### 4.3 Sample Size

This study was exploratory in nature, and as such no formal sample size calculation was conducted. Instead, the sample size was determined by the available study period.

### 4.4 Study Processes

Former patients who met the inclusion criteria were identified by nurses who performed the post‐ICU admission aftercare follow‐up calls (JAM, MH, SAG). Normally this happens 3–4 months after ICU discharge. However, due to COVID‐19, calls were sometimes made months later. During this regular call former patients were asked if they consented to being approached regarding a study on dream experiences in the ICU. They were informed that participation was also possible if they did not have dream experiences. Every couple of months the nurses sent a list of former patients who agreed to be approached to the researchers, after which they were contacted by telephone. After showing interest in participating, participant information and consent forms were provided. After a week, former patients were called to further discuss study participation and included in the study if they gave informed consent.

### 4.5 Definitions

#### 4.5.1 Delirium

The presence of delirium was determined using the Intensive Care Delirium Screening Checklist (ICDSC) score, which indicates delirium with a score of ≥ 4 [21] and/or prescription of haloperidol, as haloperidol was specifically prescribed for the treatment of delirium.

#### 4.5.2 Dream Experiences

The following question pertaining to the ICU admission was used to determine the presence of dream experiences: “Can you describe a dream that you remember the most?”. If the participant was able to describe dream content, they were classified as having had dream experiences during their ICU stay.

#### 4.5.3 Vivid Dream Experiences

Participants were asked the following question “How life‐like were the dream experiences?” to determine whether they have had vivid dream experiences. They could respond by selecting one of the following answers: “Like being awake”, “Very detailed, but clearly a dream”, “A clear story line, but few details”, “Self‐contained scenes with no clear line”, “Mainly sounds, shapes, smells, tastes or other sensory perceptions”, and “No clear content”. Participants who answered “Like being awake” were classified as having had vivid dream experiences.

#### 4.5.4 Perceptual Disturbances

Occurrence of perceptual disturbances (e.g., illusions and hallucinations) was ascertained using the following question: “Based on the fact that others did not share your perceptions or for other reasons, do you think that you experienced hallucinations (sensory experiences that were not real) during your admission?”

#### 4.5.5 Sleep Quality

Sleep quality was measured using the Richards‐Campbell Sleep Questionnaire (RCSQ) score, which is validated in critically ill patients [22]. The RCSQ consists of five questions pertaining to sleep that are scored from 0 to 10, with a higher score indicating better sleep. The total score is the sum of all sub‐scores.

#### 4.5.6 Sleep Duration

Sleep duration was recorded as the total hours of sleep during the night shift (between 10 PM and 6 AM), as reported by the attending nurse.

#### 4.5.7 Impact of Dream Experiences

The impact of dream experiences was retrospectively assessed by asking participants how these dreams affected their overall feelings about their ICU admission. Participants' responses were categorized as positive, mixed, negative or neutral, and whether these dreams continued after ICU discharge was also recorded. Additionally, at the end of the questionnaire, participants were asked if they had any additional comments; those related to the impact of the dream experiences are also included in the results.

#### 4.5.8 Disease Severity

Disease severity was recorded using the Sequential Organ Failure Assessment (SOFA) score at ICU admission. This is a widely used score, ranging from 0 to 24, that measures the severity of organ dysfunction in critically ill patients [23].

#### 4.5.9 Pain

Pain was assessed using the Numeric Rating Scale (NRS) pain score (range 0–10), respectively, a higher score indicates higher pain severity [23, 24].

#### 4.5.10 Invasive Mechanical Ventilation

The invasive mechanical ventilation duration was calculated by summing up the number of days. Ventilation was considered to have ended if weaning had started and there was no ventilation for ≥ 12 h, unless it resumed and exceeded the previous day's hours. Restarting ventilation after ≥ 48 h was considered a separate period.

### 4.6 Interview Process

Participants answered a questionnaire by telephone regarding dreams, sleep and perceptual disturbances during their ICU admission. The questionnaire was mostly structured, with only a few open questions. These questions were originally based on the interview questions from Roberts et al. (2006) but were modified and expanded to better align with the objectives of the current study. Expert and peer reviews (by seven individuals) were conducted to improve the face and content validity of the questions. If participants were unable to answer the question, the option “unknown” was selected. A formal pilot study was not conducted as this was not deemed necessary or desirable given the structured nature of the questionnaire and rigorous review. Additionally, if the participant seemed to misunderstand a question, clarification would be provided. Only one or two participants chose “unknown” for any question, aside from the question about distinguishing dreams from perceptual disturbances; for most questions, no one selected this option, which we believe supports the questionnaire's robustness. See Table S1 for the specific questions.

The calls took between 5 and 60 min depending on the content and were not recorded. Instead, notes were taken by the investigator during and directly after the interview. After the call, a copy was sent by email to the participant to allow for corrections to be made. The investigators were aware of the participant's name, age, sex, LOS and contact information but did not know clinical details at that point. Participants were called by AEH (a PhD student in medicine) or students who had received the same instructions and had a similar background.

### 4.7 Data Collection

In addition to the questionnaire, patient group data were retrieved from electronic health records (see Table S2 for an overview of collected variables) to describe the study population and determine potential factors related to the occurrence of (vivid) dream experiences. Data collection at the time of admission was done by nurses as part of the daily usual care on the unit. To deal with missing data, daily averages of scores were taken, and then those daily averages were averaged over the entire admission duration. This approach also aligns with our primary focus on outcomes across the full admission period rather than on specific daily events. If data for a specific variable were not available for all participants, the alternative sample size (*n*) is indicated for that variable. For group comparisons, only factors that did not have to be aggregated over the admission were included: age, sex, LOS, time since ICU discharge, SOFA at admission, COVID‐19 positivity at admission, elective/non‐elective admission, intubation during admission (yes/no) and overall subjective sleep quality.

Medication use was documented for opioids, anaesthetics, benzodiazepines, corticosteroids, antipsychotics, antidepressants and antiepileptic drugs. Whether it was used at all and the percentage of the LOS (number of days prescribed/LOS) it was used were recorded.

The ICU from which former ICU patients were recruited has an adjacent Intermediate Care Unit (IMCU) as part of the same ward. For the purpose of this study, patients who were transferred between the ICU and IMCU during their admission were regarded as a single ICU admission. In cases of multiple admissions meeting the inclusion criteria, only the latest admission applied, unless the patient was readmitted within a week for the same reason, in which case the admittance days were combined.

### 4.8 Data Analysis

The primary objective was determining (1) the extent of (vivid) dream experiences in the ICU. The occurrence of these dream experiences was assessed based on participants' responses regarding their ICU admission at the time they completed the questionnaire. All statistical analyses were performed using SPSS version 25, with a two‐tailed significance level of α = 0.05. Normally distributed continuous data are presented as mean and standard deviation (SD). Non‐normally distributed continuous data are presented as median and interquartile range (IQR). Depending on the distribution, the unpaired *T*‐test or the Mann–Whitney U test were used to analyse the significance of differences in continuous variables. Categorical data are presented as counts and percentages of the total. The Chi‐square test was applied to test for differences when comparing categorical data. In case of < 5 observations within a contingency table cell, Fisher's Exact Test (FET) was used.

The following methods were applied to determine the secondary objectives regarding (2) dream content, (3) impact, (4) associated factors and (5) the overlap between dreams, perceptual disturbances and delirium. To address the second objective, descriptions of dream experiences are reported qualitatively. Recurring descriptions are reported as a percentage of all included participants and of the total number of dreams of the participants. The third objective, the impact of (vivid) dream experiences, was assessed by reporting the numbers and percentages of responses to relevant questions, highlighted in the ‘Definitions’ section above. To determine the fourth objective, potential associated factors, differences in variable outcomes were assessed between participants with and without reported dream experiences, and between those with and without vivid dream experiences. The fifth objective, exploring the overlap between delirium, dream experiences and perceptual disturbances, was investigated. Data analysis involved determining the prevalence of delirium and perceptual disturbances and identifying the extent of overlap between these conditions. Participants' ability to differentiate between dreams and perceptual disturbances and the difference in occurrence of vivid dreams between a subgroup with perceptual disturbances and the total population was assessed.

#### 4.8.1 Dream Content Analysis

A content analysis was performed based on the method suggested by Graneheim and Lundman (2004) in which text units are categorized into themes, categories and sub‐categories [25]. Two investigators (AEH and CR) independently reviewed interview notes focusing on participants' most notable dream content, based on the following interview question: “Can you describe a dream that you remember the most?”. The dream content was then organized into distinct themes, not defined beforehand. Initially, an overall understanding of the participants' dream content was obtained by examining the text overview of the notes of all participant dream experience content descriptions. Subsequently, a thorough re‐reading was performed, during which text units were condensed into an underlining meaning and categorized into themes. Additionally, the investigators separately assessed the overall emotional tone of each dream, categorizing them as positive, neutral or negative. Following individual data review, the two investigators collaborated to determine the final tone classification for each dream and to establish the final theme, categories and sub‐categories based on consensus after comparing their respective classifications.

When referring to a specific participant, their subject number (p1‐p80) is mentioned.

### 4.9 Ethical Considerations

The study was conducted per the Helsinki Declaration as revised in 2013 and in accordance with local statutory requirements of the medical ethical committee of Leiden, The Hague and Delft (registration number N21.058). It was also reviewed and approved by the scientific committee of the intensive care unit on 11 March 2021. All participants provided written informed consent for participation, data collection and publication.

## 5 Results

### 5.1 Study Population

**FIGURE 1:**
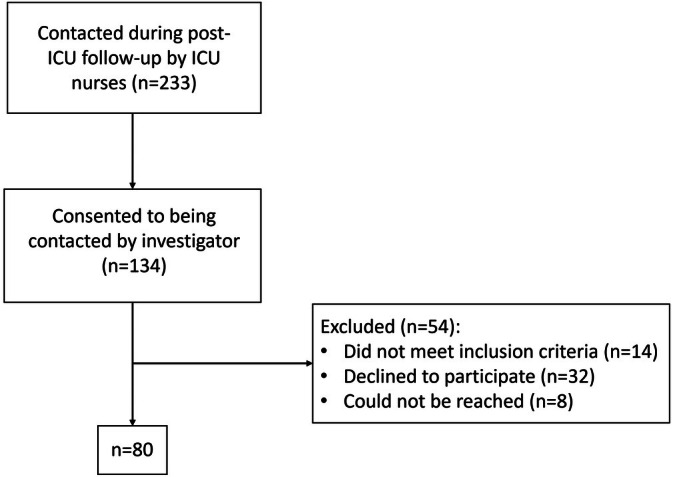
Inclusions.

Of the 233 former ICU patients contacted during regular post‐discharge follow‐up procedures, 134 consented to being contacted. Of those, 80 could be included (see Figure 1).

The median age of the participants was 61 (53–68), 71.3% was male (see Table 1). The median LOS and the median time after ICU discharge were 10 and 273 days, respectively. One participant was admitted 3 days, instead of ≥ 4 days, and was included due to being COVID‐19 positive. Excluding this patient did not significantly alter the results (data not shown).

**TABLE 1:** Participant characteristics (*n* = 80).

	*n* = 80
Demographics	
Age in years	61 (53–68)
Gender, *n* (% male)	57 (71.3)
Admittance characteristics	
LOS in days	10 (7–19)
Time in days after discharge	273 (218–310)
Elective admission, *n* (%)	14 (17.5)
SOFA at admission, mean ± SD	7.5 ± 3.1
Average NRS pain score	0.25 (0.0–1.0), *n* = 77^a^
Maximum NRS pain score	2.0 (0.0–5.5), *n* = 77^a^
COVID‐19 positivity, *n* (%)	43 (53.8)
Mechanical ventilation, *n* (%)	72 (90.0)
Mechanical ventilation in days	7 (2–10)

*Note:* If not specified median and interquartile range (IQR) are reported.

Abbreviations: COVID‐19, coronavirus disease 2019; LOS, length of stay; NRS, numerical rating scale; SOFA, sequential organ failure assessment.

^a^ No pain scores were taken for 3 participants.

### 5.2 Medication Use During ICU Admission

An overview of medication used by participants in the ICU is shown in Table S3. Opioids, anaesthetics, benzodiazepines, corticosteroids and antipsychotics were prescribed in the majority of cases (all in more than 50% of participants). Anaesthetics (the most reliable indicator of sedation in our data) were not administered for a median of 43% of the ICU admission duration.

### 5.3 Sleep in the ICU

**FIGURE 2:**
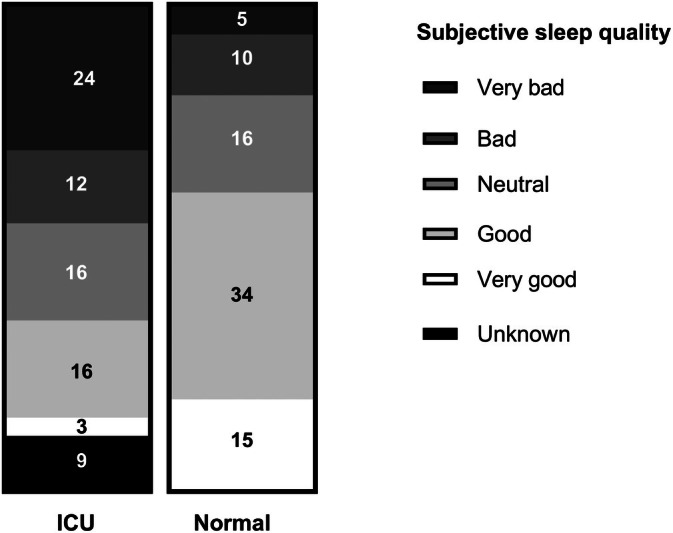
Self‐reported sleep quality during and outside of the intensive care unit (ICU) admission.

The average RCSQ score was 22.9 ± 9.1 out of a maximum of 50 (*n* = 56). The average night‐time (between 10 PM and 6 AM) sleep duration was 4.0 ± 1.4 h (*n* = 58). Normal/regular self‐reported sleep was generally better than the sleep during the ICU admission (see Figure 2).

### 5.4 Dream Experiences in the ICU

**FIGURE 3:**
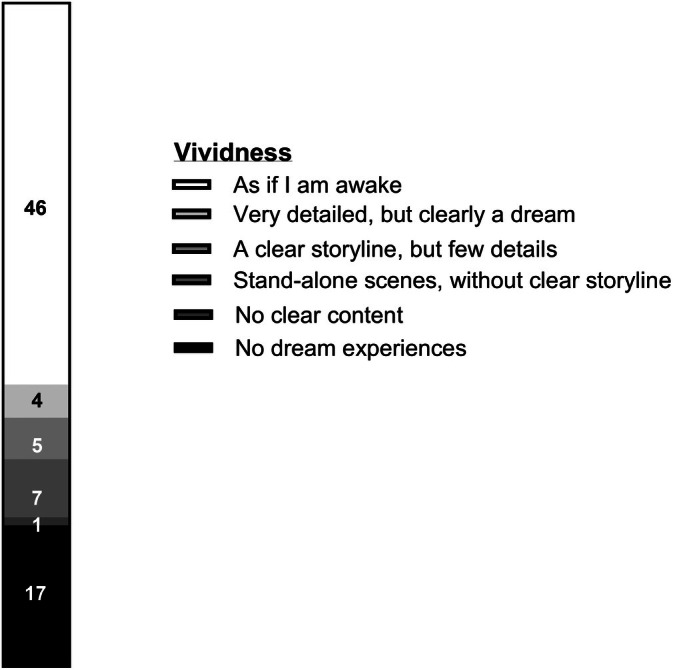
Intensive care unit stay dream vividness according to participants (*n* = 80).

Seventy‐eight percent (63/80) reported that they had dream experiences during their ICU stay. Of these 63 participants, 73.0% described their dream experiences as vivid (see Figure 3) and 73.0% said they had auditory sensations during dreams. Somatic sensations (i.e., perception of tactile stimuli) during dreams were experienced by 49.2% of the participants with dream experiences.

Within the group of participants with dream experiences, 49.2% said they generally experienced negative feelings about these dreams in the context of their overall ICU admission; 11.1% had positive feelings, 19.0% mixed feelings and 20.6% neutral feelings. A respective 79.4% and 90.5% indicated that the frequency and content of their dreams in the ICU differed from their dreams outside of the ICU. Additionally, 28.6% reported having similar dream experiences post‐ICU discharge, while only 6.3% reported having had such experiences before their ICU admission.

### 5.5 Content of Dream Experiences

The participants who recollected dream experiences (*n* = 63) described one or more experiences that were most notable/memorable to them (median of 2 dreams, maximum 5, total *n* = 127). Seventy‐three percent of all described dreams had a negative content, 22.2% had positive content and 42.9% had neutral content. Five participants (7.9%) described having had a “continuous dream” (p2, p25, p26, p55, p68). Notably, these participants were all delirious at some point during their ICU stay. Dream content was mostly related to the hospital stay (49.2%), helplessness (47.6%), family/friends/acquaintances (44.4%) and death (36.5%). Participants also frequently dreamt about lying down in a bed, on a platform, or in a vehicle (25.4%) or about various conflict situations (25.4%) (see Table S6 for a complete overview of dream themes and sub‐themes).

The most common dream themes were often present within the same dream. In some cases, participants described that they were abandoned by hospital staff and/or family and friends in their dreams, increasing their feeling of helplessness. One participant described dreaming about an interaction with a nurse during which the nurse said, “I'm not helping you anymore, you can sort it out with my colleague”, abandoning the patient in a dark, cold basement (p27). Most participants (69.8%) believe that their dream experiences were substantively related to experiences during their ICU admission. For example, three participants reported dreams in which they were underwater or just below the surface and unable to escape (p11, p29, p57). One of them suggested that this could be linked to his breathing difficulties during the ICU stay. Two out of three were admitted to the ICU due to cardiopulmonary‐related problems, requiring supplemental oxygen.

### 5.6 Impact of ICU Dream Experiences

Some participants found their dreams to be quite pleasant or believed that they mitigated the intensity of their experience in the ICU (p8, p41, p65). However, multiple participants reported a negative effect from the dreams even after waking up. Fifty‐four percent of participants stated the dreams continued to impact them after awakening. To illustrate this, one participant dreamed that someone was throwing his bones into a large fire. Upon waking he said to his wife, “This dream has prophetic value. I am going to die!”. Another participant mentioned difficulties sleeping after being back at home, due to the traumatic nature of his dreams during his ICU stay (p47). Others remained convinced that the events they had dreamt about had really happened, only realizing later that they dreamt it (p11, p21, p42, p49, p54, p63). A participant dreamt that her husband was angry with hospital staff because they would not allow her to be discharged. Upon waking she was under the impression that her husband was still angry (p54). Another participant had a dream in which a small man wearing a yellow vest groped him. It took over a week to reconcile with the fact that this event could not have taken place in reality (p80). Another example is of a participant who said he dreamed that a nurse was mean to him, after he woke up he tried to kick her (p63).

### 5.7 Participant Perspectives on Coping With ICU Dream Experiences

When concluding the dream experience part of the interview, participants were asked if they had any additional comments regarding their ICU admission in general, and 11 of them did. Two participants said they had been relieved to hear that other ICU patients had similar experiences during their admission (p44, p49). Three participants said that having someone (familiar) present to support them or explain what was happening with them and around them would have been beneficial (p16, p69, p79). Four expressed interest in speaking to the nurses and/or physicians who had cared for them in the ICU or returning to the ICU (p6, p13, p14, p74). Two others mentioned that they had talked about their dream experiences with a psychiatrist after discharge and that they believe this has helped them (p11, p39).

### 5.8 Factors Associated With Vivid Dream Experiences

Participants with vivid dream experiences (*n* = 46) were younger and had a longer length of stay than those who did not report vivid dream experiences (*n* = 34, all *p* < 0.05, see Table S4). While non‐significant, a higher number among those with vivid dream experiences had been intubated compared to the group without vivid dream experiences (*p* = 0.066). Sleep outcomes, SOFA and the percentage of elective admissions did not differ significantly between those with and without vivid dream experiences.

Participants who reported having had dream experiences in general (*n* = 63) had a longer admittance duration than those who did not report this (*n* = 17, median of 13.0 versus 7.0 days, *p* < 0.001, see Table S5) and had been intubated more often (95.2 versus 70.6%, FET, *p* = 0.009).

There were no significant differences in reported vivid dream experiences or dream experiences in general between participants admitted with COVID‐19 and those without (respectively, 65.1% versus 48.6%, *p* = 0.175 and 83.7% versus 73.0%, *p* = 0.281).

### 5.9 Partial Overlap Between Reported Perceptual Disturbances, Dream Experiences and Delirium

The majority (62.5%) of participants had a positive delirium score and/or used haloperidol at any time during their admission (see Table 2). In addition to that, a substantial number of participants (50.0%) reported experiencing perceptual disturbances. There was a large overlap between those with delirium and those experiencing perceptual disturbances, as 70.0% of the perceptual disturbances subgroup had a confirmed delirium. Fifty‐five percent could not (with certainty) differentiate dreams from perceptual disturbances. Notably, the occurrence of perceptual disturbances was not noticed during the ICU/IMCU stay by the attending physicians or nurses.

**TABLE 2:** Delirium related outcomes in the total group (*n* = 80) and within a subgroup reporting perceptual disturbances during their intensive care unit stay (*n* = 40).

	All participants (*n* = 80)	Perceptual disturbances subgroup (*n* = 40)	Test statistic	*p*‐value
Delirium				
ICDSC+	27 (39.7), *n* = 68	15 (44.1), *n* = 34	χ^2^ = 0.182	0.831
ICDSC+ and haloperidol use	50 (62.5)	28 (70.0)	χ^2^ = 0.659	0.543
Dream experiences				
All	63 (78.8)	34 (85.0)	χ^2^ = 0.672	0.470
Vivid	46 (57.5)	31 (77.5)	χ^2^ = 4.639	0.043*
Differentiation dreams vs. perceptual disturbances	—	18 (45.0)		—
Type of perceptual disturbances				
Visual	—	36 (90.0)		—
Auditory	—	20 (50.0)		—
Somatic	—	11 (27.5)		—
Unknown	—	2 (5.0)		—
Timing perceptual disturbances				
During waking or falling asleep	—	12 (30.0)		—
When awake	—	10 (25.0)		—
When awake and when falling asleep	—	11 (27.5)		—
Unknown	—	7 (17.5)		—

*Note:* Count and percentage are reported for all variables. The *n* is mentioned when not 80 or 40, respectively.

Abbreviation: ICDSC+, intensive care delirium screening checklist score ≥ 4.

* means Statistically significant.

The percentage of reported dream experiences was higher in the perceptual disturbances subgroup than in all participants (85% vs. 78.8%, *p* = 0.470). This was also the case for vivid dream experiences (77.5 vs. 57.5%, *p* = 0.043).

## 6 Discussion

Vivid dream experiences are frequently retrospectively reported by participants. Even after several months, most participants had memories of very specific, mostly negative, dream experiences from their IC stay, reflecting the impact of these experiences. The most common dream themes were related to the ICU admission itself. A number of factors may be related to the vividness of the reported dream experiences, including age, length of stay and mechanical ventilation. Half of the participants reported perceptual disturbances, but many of them could not distinguish these from dream experiences. There is a large overlap between those with perceptual disturbances and those who, with certainty, had a delirium.

### 6.1 Occurrence of Dream Experiences and Associated Factors

The occurrence of self‐reported dream experiences was high (79% after a median of 9 months). Roberts et al. [4] performed interviews 12–18 months after ICU discharge and reported 74% of patients who had ICU stays of at least 3 days experienced dreams. Another study with 41 participants who had ICU stays of at least 36 h found a dream experience prevalence of 44% after up to 2 years post‐discharge [8]. Previous research does support an association between reported dream experiences and length of stay [4, 8, 9], and with invasive mechanical ventilation [4, 8], supporting our findings. A connection between age and the occurrence of dream experiences was not found in prior studies. More, preferably prospective and longitudinal, research is needed to gain further insights into the various factors affecting the occurrence of dream experiences in the ICU.

### 6.2 Dream Content

While dream content in the ICU generally differs from the general population [26, 27], some typical themes like being chased, attacked, trapped or attempting something repeatedly were mentioned. For instance, a participant described recurring dreams of battling a “Corona [virus] monster”, failing twice and finally defeating it, then consuming the monster (p15). This exemplifies a common theme observed in reported dreams, specifically their connection to the ICU stay.

Differences in dream content between our study population and the general population could be caused by the different study population and methodology, as we did not use the Typical Dream Questionnaire [26, 27, 28], but asked questions focused on the specific situation of the participants. Participants described their most memorable dreams after an extended period of time, potentially impacting recall and introducing selection bias and skewing the descriptions away from more “typical”, less memorable dream themes [13].

Many participants considered the content of their dreams to be related to the ICU admission itself. These dreams often had a negative content (helplessness, darkness, not being heard or seen or being lost). Similarly, another study found that 60.9% of participating ICU patients experienced “scary” dreams [4] or dreams associated with negative emotions [4, 8, 9]. Several studies hypothesize that dreams have an adaptive function in emotion regulation by preparing the individual to react to and resolve emotional conflicts, achieving emotional mastery [29, 30]. The heightened amygdala activity during rapid eye movent (REM) sleep, regulating responses to stress, might explain negative or scary dream content [30, 31]. Although the proportion of time spent in REM sleep decreases in the ICU, the increased sleep fragmentation people experience in the ICU could result in more frequent awakenings during REM sleep, resulting in a greater recall of negative dreams [32, 33]. Additionally, dreams tend to be a continuation of experiences that people are likely to have in the waking world [34], perhaps explaining the high prevalence of ICU‐related dream content. Overall, our findings suggest that ICU patients experience dreams that are often related to their admission and frequently have a negative content. These results contribute to our knowledge regarding the emotional and psychological experiences of ICU patients and could provide a starting point for further research in this area.

### 6.3 Relationship Between Dream Experiences and Perceptual Disturbances

We found a large overlap between reported perceptual disturbances, dream experiences and delirium. A multicentr cohort study found a (non‐significant) trend towards increased prevalence of dreaming among those with a delirium compared to non‐delirious patients [8]. Similarities have previously been noted between characteristics of dreams and delirium [35], hallucinations [15] and even psychosis [36]. Possible explanations for why many patients reported perceptual disturbances, even though not all had an established delirium, could be unnoticed delirium, medication side effects and/or the effects of prolonged sleep deprivation without delirium being present (yet) [37, 38, 39]. The presence of hypnagogic and/or hypnopompic hallucinations (which occur during transitional states between wakefulness and sleep) could be an additional explanation [40, 41]. Furthermore, many ICU patients also sleep during the day, which could make it more difficult to distinguish between experiences during wakefulness and sleep. The findings of the current study do not clarify the exact relationship between dream experiences, perceptual disturbances and delirium. These findings do, however, illustrate that the distinction can be difficult to make for both the people having these experiences as well as the researchers studying them. In future studies, it would be interesting to further elucidate the differentiation between dreams, delirium and perceptual disturbances.

### 6.4 The Post‐Discharge Consequences of Dream Experiences

Some patients report experiencing similar vivid, strange and negative dreams after discharge from the ICU. This is consistent with findings from Roberts et al. (2004) who reported a recurrence of dream experiences in 22% [4]. Previous research has also shown that fear associated with such experiences can be partially mitigated by care actions and support by relatives [5]. However, patients usually do not inform others of unreal experiences [42]. In this study, several participants indicated that knowing others have similar experiences, receiving support from familiar individuals or discussing their experiences with their health care providers would be beneficial. These findings emphasize the importance of monitoring and addressing patients' dream experiences during and after ICU admission.

## 7 Limitations

Limitations include a potential for selection bias, as patients with more dream experiences may have been more likely to participate. Additionally, only patients with a longer admission duration participated, which may have impacted the generalizability to the ICU population. The sample size is larger than comparable studies but may still miss smaller differences. Moreover, only 17 participants reported no dream experiences, limiting the significance of comparisons between groups. Future prospective studies with a more targeted research question and larger sample sizes are needed to draw more definitive conclusions about differences between those with and without reported dream experiences. The data are self‐reported and the follow‐up time was around 9 months, which may have resulted in recall bias potentially leading to overemphasis on more extreme dream experiences and underestimating the prevalence of dream experiences in general. However, given the already high prevalence of dream reports in our study, an even higher prevalence would further strengthen our conclusions. Furthermore, by asking participants to describe notable dream(s) this could have prompted them to recall extremely negative or positive experiences. The framing of this question should be taken into account when interpreting the results. Additionally, relying on note‐taking may have restricted the depth of qualitative data and introduced variability in how responses were captured. Future studies using recorded interviews could provide more in‐depth characterization of patient experiences. Lastly, the reported variables were aggregated across the ICU stay (i.e., means and medians of the whole admission were reported instead of per day). A longitudinal prospective design may be necessary to examine direct impacts of factors such as medication use and delirium on dream experiences.

## 8 Implications and Recommendations for Practice

This study highlights the need for targeted interventions regarding vivid negative dream experiences in the ICU. Nurses are in a key position to routinely assess for such experiences. Offering psychological support, normalizing these phenomena and informing patients and families about the potential for these dreams could help to reduce emotional distress. Further, creating a supportive environment that encourages patients to share their experiences may enhance recovery and emotional well‐being post‐discharge.

## 9 Conclusion

Dream experiences were common and frequently associated with negative emotions among ICU patients with a relatively long length of stay in the ICU. Dreams were frequently vivid. Younger age and longer length of stay are potentially related to more occurrences of vivid dream experiences. Dream content often pertained to the ICU admission, with feelings of helplessness being a common theme. Half of the participants reported perceptual disturbances, with a substantial overlap between these experiences and delirium. Many could not distinguish perceptual disturbances from dream experiences. Further research is needed on dream experiences in ICU patients, including the extent of their impact during the ICU admission, potential impact on post‐ICU mental well‐being, causes and ways to minimize negative experiences.

## Ethics Statement

The study was conducted per the Helsinki Declaration as revised in 2013 and in accordance with local statutory requirements of the medical ethical committee of Leiden, The Hague and Delft (registration number N21.058). It was also reviewed and approved by the scientific committee of the Intensive Care Unit on 11 March 2021. All participants provided written informed consent for participation, data collection and publication.

## Conflicts of Interest

The authors declare no conflicts of interest.

## Supporting information


**Table S1.** Questionnaire. *Answer option “I don’t know” was selected if the participant was unable to answer.
**Table S2.** Collected variables. ICU; Intensive Care Unit, COVID‐19; Coronavirus disease 2019, SOFA; Sequential Organ Failure Assessment, NRS; numerical rating scale, RCSQ; Richards‐Campbell Sleep Questionnaire.
**Table S3.** Medication use. If not specified median and interquartile range (IQR) are reported.
**Table S4.** Differences between participants who report life‐like dream experience content and those who do not. If not specified median and interquartile range (IQR) are reported. SOFA; Sequential Organ Failure Assessment, COVID‐19; Coronavirus disease 2019.
**Table S5.** Differences between participants who report dream experience content and those who do not. If not specified median and interquartile range (IQR) are reported. SOFA; Sequential Organ Failure Assessment, COVID‐19; Coronavirus disease 2019.
**Table S6.** Dream content frequency (*n* = 63).

## Data Availability

The data that support the findings of this study are available from the corresponding author upon reasonable request.
