# Supplementary material for: Dream Experiences During Intensive Care Unit Stay: Occurrence, Content, Vividness and Associated Factors
Source: Nurs Crit Care. 2025 Jul 2;30(4):e70106. doi: 10.1111/nicc.70106 (PMC12222043; doi:10.1111/nicc.70106)
Supplement: Supplementary file 1 — Table S1. Questionnaire. *Answer option “I don’t know” was selected if the participant was unable to answer. Table S2. Collected variables. ICU; Intensive Care Unit, COVID‐19; Coronavirus disease 2019, SOFA; Sequential Organ Failure Assessment, NRS; numerical rating scale, RCSQ; Richards‐Campbell Sleep Questionnaire. Table S3. Medication use. If not specified median and interquartile range (IQR) are reported. Table S4. Differences between participants who report life‐like dream experience content and those who do not. If not specified median and interquartile range (IQR) are reported. SOFA; Sequential Organ Failure Assessment, COVID‐19; Coronavirus disease 2019. Table S5. Differences between participants who report dream experience content and those who do not. If not specified median and interquartile range (IQR) are reported. SOFA; Sequential Organ Failure Assessment, COVID‐19; Coronavirus disease 2019. Table S6. Dream content frequency (n = 63). [file NICC-30-0-s001.docx]

**Supplementary material**

Table S1: Questionnaire

| **Question** | **Answer options** |
| --- | --- |
| Can you describe a dream [you had during your ICU stay] that you remember the most? | Open question |
| Did the dream experiences in the ICU differ in content from the dream experiences usual for you outside the ICU environment? | Yes/no |
| Did the dream experiences in the ICU differ in terms of frequency (how often they occur) from the dream experiences outside the ICU environment that were usual for you? | Yes/no |
| Did you continue to have these dream experiences after you were discharged from the ICU? | Yes/no |
| Does the content of your dream experiences relate to experiences you had in the ICU? (yes/no) | Yes/no |
| What feelings did the dream experiences evoke for you when you consider the entire ICU stay? | negative, positive, neutral, mixed |
| Did the dream experiences affect your functioning during the time you were awake? | Yes/no |
| How life-like were the dream experiences? | Like being awake/ Very detailed, but clearly a dream/ A clear story line, but few details/ Self-contained scenes with no clear line/ Mainly sounds, shapes, smells, tastes or other sensory perceptions/ no clear content |
| During your dreams, did you only see things or did you also hear and feel things? | Only seeing/ Also hearing/ Also feeling/ Also hearing and feeling |
| How would you rate your normal sleep quality? | Poor/neutral/good |
| How would you rate your sleep quality during the intensive care unit stay? | Poor/neutral/good |
| Is there anything left unsaid that you would like to share? | Open question |
| Due to the fact that others did not share your perceptions or by otherwise reasoning, do you think you hallucinated (had sensory experiences that were not real) during your admission? | Yes/no |
| Were the hallucinations and real-life dream experiences distinguishable from each other? | Yes/no |

*Answer option “I don’t know” was selected if the participant was unable to answer

Table S2: Collected variables

| Category | Items |
| --- | --- |
| Demographics | Age  Sex |
| General medical information | Date of ICU admission and ICU discharge  Duration of ICU stay  Relevant comorbidities (such as sleep disorders)  SOFA score at admission  Reason for admission  Pain scale scores (Numerical Rating Scale)  COVID-19 positivity  Mechanical ventilation details during ICU stay  Medication use during ICU stay  Sleep scores (RCSQ score and sleep duration during the night shift)  Presence of delirium during ICU stay |

*Abbreviations: ICU; Intensive Care Unit, COVID-19; Coronavirus disease 2019, SOFA; Sequential Organ Failure Assessment, NRS; numerical rating scale, RCSQ; Richards-Campbell Sleep Questionnaire

Table S3: Medication use

| n=80 |  |
| --- | --- |
| Medication use | |
| Opioids, n (%) | 75 (93.8) |
| - % of time used | 70.0 (50-81) |
| Anesthetics, n (%) | 73 (91.3) |
| - % of time used | 57.1 (31-74) |
| Benzodiazepines, n (%) | 72 (90.0) |
| - % of time used | 43.9 (25-76) |
| Corticosteroids, n (%) | 62 (77.5) |
| - % of time used | 87.9 (51-100) |
| Antipsychotics, n (%) | 44 (55.0) |
| - % of time used | 21.6 (11-37) |
| Antidepressants, n (%) | 4 (5.0) |
| - % of time used | 85.7 (46-100) |
| Antiepileptic drugs, n (%) | 4 (5.0) |
| - % of time used | 81.3 (40-93) |

*Notes: if not specified median and interquartile range (IQR) are reported

Table S4: Differences between participants who report life-like dream experience content and those who do not

| n=80 | Life-like dream experience, n=46 | Unknown/no life-like dream experience, n=34 | Test statistic | P-value |
| --- | --- | --- | --- | --- |
| Demographics | | | | |
| Age, mean ±SD | 58 (51-63) | 64 (59-71) | H=533.000 | .015* |
| Gender, n (% male) | 34 (73.9) | 23 (67.6) | χ^2^=.375 | .620 |
| Admittance characteristics | | | | |
| Length of stay in days | 12.5 (9-20) | 8 (6-15) | U=1017.000 | .022* |
| Time in days after discharge | 274 (221-310) | 262 (207-313) | U=836.000 | .599 |
| Elective admission, n (%) | 5 (10.9) | 9 (26.5) | χ^2^=3.296 | .082 |
| SOFA at admission | 7.5 (5-9) | 7.0 (6-9) | U=727.000 | .590 |
| COVID-19 positivity at admission, n (%) | 28 (60.9) | 15 (44.1) | χ^2^=2.207 | .175 |
| Mechanical ventilation, n (%) | 44 (95.7) | 28 (82.4) | FET | .066 |
| During ICU admission - self reported sleep quality, count/n (%) | | | | |
| Very bad | 17/46 (37.0) | 7/34 (20.6) | FET | .368 |
| Bad | 5/46 (10.9) | 7/34 (20.6) | - | - |
| Neutral | 10/46 (21.7) | 6/34 (17.6) | - | - |
| Good | 9/46 (19.6) | 7/34 (20.6) | - | - |
| Very good | 2/46 (4.3) | 1/34 (2.9) | - | - |
| Unknown | 3/46 (6.5) | 6/34 (17.6) | - | - |
| Outside of ICU admission - self reported sleep quality, count/n (%) | | | | |
| Very bad | 5/46 (10.9) | 0/34 (0.0) | FET | .233 |
| Bad | 5/46 (10.9) | 5/34 (14.7) | - | - |
| Neutral | 11/46 (23.9) | 5/34 (14.7) | - | - |
| Good | 17/46 (37.0) | 17/34 (50.0) | - | - |
| Very good | 8/46 (17.4) | 7/34 (20.6) | - | - |

*Notes: if not specified median and interquartile range (IQR) are reported

*Abbreviations: SOFA; Sequential Organ Failure Assessment, COVID-19; Coronavirus disease 2019

Table S5: Differences between participants who report dream experience content and those who do not

| n=80 | Dream experience, n=63 | Unknown/no dream experience, n=17 | Test statistic | P-value |
| --- | --- | --- | --- | --- |
| Demographics | | | | |
| Age | 60 (53-64) | 67 (54-72) | U=388.500 | .084 |
| Gender, n (% male) | 46 (73.0) | 11 (64.7) | FET | .552 |
| Admittance characteristics | | | | |
| Length of stay in days | 13.0 (9.0-19.0) | 7.0 (4.5-8.5) | U=850.500 | <.001* |
| Time in days after discharge | 276 (222-311) | 238 (179-293) | U=677.500 | .095 |
| Elective admission, n (%) | 10 (15.9) | 4 (23.5) | FET | .482 |
| SOFA at admission | 8.0 (6.0-10.0) | 7.0 (5.5-8.5) | U=600.000 | .445 |
| COVID-19 positivity at admission, n (%) | 36 (57.1) | 7 (41.2) | χ^2^=1.373 | .281 |
| Mechanical ventilation, n (%) | 60 (95.2) | 12 (70.6) | FET | .009* |
| During ICU admission – self reported sleep quality, count/n (%) | | | | |
| Very bad | 20/63 (31.7) | 4/17 (23.5) | FET | .839 |
| Bad | 9/63 (14.3) | 3/17 (17.6) | - | - |
| Neutral | 13/63 (20.6) | 3/17 (17.6) | - | - |
| Good | 13/63 (20.6) | 3/17 (17.6) | - | - |
| Very good | 2/63 (3.2) | 1/17 (5.9) | - | - |
| Unknown | 6/63 (9.5) | 3/17 (17.6) | - | - |
| Outside of ICU admission - self reported sleep quality, count/n (%) | | | | |
| Very bad | 5/63 (7.9) | 0/17 (0.0) | FET | .596 |
| Bad | 8/63 (12.7) | 2/17 (11.8) | - | - |
| Neutral | 14/63 (22.2) | 2/17 (11.8) | - | - |
| Good | 24/63 (38.1) | 10/17 (58.8) | - | - |
| Very good | 12/63 (19.0) | 3/17 (17.6) | - | - |

*Notes: if not specified median and interquartile range (IQR) are reported

*Abbreviations: SOFA; Sequential Organ Failure Assessment, COVID-19; Coronavirus disease 2019

Table S6: Dream content frequency (n=63)

| **Theme** | **Sub-theme** | | **Patient participant and dream number** | **Dreams aggregated per patient (n=63)** | | | **All dreams (n=127)** | | |
| --- | --- | --- | --- | --- | --- | --- | --- | --- | --- |
|  |  |  |  | **Theme** | **Sub-theme** | | **Theme** | **Sub-theme** | |
| Death/dying  n (%) | Physical passage to death | | D3, D10, D35.1, D49.2, D65.2 | 23 (36.5) | 5 (7.9) | | 27 (21.3) | 5 (3.9) | |
|  | Seeing someone who is deceased alive | | D36.1, D36.2, D65.1, D67.1 |  | 3 (4.8) | |  | 4 (3.1) | |
|  | Participants’ own death | Imminent/potential death | D2.1, D4.1, D29, D31.2, D39.2, D47.4, D49.2 |  | 14 (22.2) | 7 (11.1) |  | 14 (11.0) | 7 (5.5) |
|  |  | Actual death | D6.2, D14.1, D15.1, D19, D25, D55, D67.3 |  |  | 7 (11.1) |  |  | 7 (5.5) |
|  | Someone else’s death | Imminent/potential death | D16, D21.5 |  | 8 (12.7) | 2 (3.2) |  | 8 (6.3) | 2 (1.6) |
|  |  | Actual death | D35.1, D42, D48.2, D49.2, D67.4, D72.2 |  |  | 6 (9.5) |  |  | 6 (4.7) |
| Travel  n (%) | In vehicle | | D2.3, D9, D17.1, D21.3, D30, D35.2, D41, D54.1, D58.3, D64, D66.3, D67.2, D68, D69.1, D69.3, D79.1 | 20 (31.7) | 15 (23.8) | | 22 (17.3) | 16 (12.6) | |
|  | Going from one location to another | | D2.3, D9, D17.1, D21.1, D41, D50.1, D64, D69.1, D69.3 |  | 8 (12.7) | |  | 8 (6.3) | |
|  | Time travel | | D10, D38.2, D63.2 |  | 3 (4.8) | |  | 3 (2.4) | |
|  | Being lost | | D9, D17.2, D51.2 |  | 3 (4.8) | |  | 3 (2.4) | |
| Helplessness  n (%) | Being lost | | D9, D17.2, D51.2 | 30 (47.6) | 3 (4.8) | | 44 (34.6) | 3 (2.4) | |
|  | Being stuck somewhere | | D2.1, D4.1, D10, D11.2, D17.1, D21.1, D25, D26.1, D29, D32.1, D33.2, D38.1, D38.2, D44.2, D47.1, D47.2, D47.3, D49.1, D50.1, D50.2, D57, D58.3, D61, D66.1, D66.3, D68, D69.5, D71.1, D73.1, D80.1 |  | 25 (39.7) | |  | 30 (23.6) | |
|  | Not being seen or heard | | D2.1, D2.2, D27.2, D33.2, D44.3, D47.3, D50.1, D51.1, D51.3, D58.3, D71.1, D73.1 |  | 10 (15.9) | |  | 12 (9.4) | |
|  | Facing an obstacle/barrier that cannot be overcome | | D4.2, D27.1, D73.2 |  | 3 (4.8) | |  | 3 (2.4) | |
|  | No control over one’s body or actions | | D25, D44.2, D49.1, D50.2, D54.1, D63.3, D69.5, D80.2 |  | 8 (12.7) | |  | 8 (6.3) | |
| Conflict  n (%) | Own involvement | Escaping from someone | D15.1, D33.2, D47.2, D49.1 | 16 (25.4) | 10 (15.9) | 4 (6.3) | 18 (14.2) | 12 (9.4) | 4 (3.1) |
|  |  | Physical abuse | D15.1, D17.1, D33.2, D39.1, D44.2, D47.4 |  |  | 6 (9.5) |  |  | 6 (4.7) |
|  |  | Physically fighting | D4.2, D15.1 |  |  | 2 (3.2) |  |  | 2 (1.6) |
|  |  | Argument | D17.1, D27.2, D63.2, D63.3, D63.4 |  |  | 5 (7.9) |  |  | 5 (3.9) |
|  | No involvement from participant | Physical abuse | D6.1 |  | 4 (6.3) | 2 (3.2) |  | 5 (3.9) | 1 (0.8) |
|  |  | Argument | D37, D54.2, D67.1 |  |  | 2 (3.2) |  |  | 3 (2.4) |
|  |  | Riots | D48.2 |  |  | 1 (1.6) |  |  | 1 (0.8) |
| Participants’ social circle  n (%) | Partner | | D2.2, D2.3, D6.1, D21.3, D21.5, D26.2, D32.1, D40.2, D44.1, D54.2, D61, D70.2, D72.2 | 28 (44.4) | 11 (17.5) | | 40 (31.5) | 13 (10.2) | |
|  | Family | | D2.2, D2.3, D6.2, D16, D32.2, D33.1, D37, D39.2, D40.1, D42, D47.1, D57, D61, D63.1, D65.1, D65.2, D65.3, D69.4, D70.2, , D71.1, D71.2, D72.1 |  | 18 (28.6) | |  | 22 (17.3) | |
|  | Friends | | D2.1, D13, D40.1, D70.1, D79.1, D79.2 |  | 5 (7.9) | |  | 6 (4.7) | |
|  | Acquaintances | | D4.1, D6.1, D33.1, D37, D40.2, D43.1, D44.1, D51.2, D55, D72.1, D79.1 |  | 12 (19.0) | |  | 12 (9.4) | |
| Hospital-related  n (%) | Hospital personnel | | D24, D25, D27.2, D33.2, D37, D39.2, D43.1, D47.3, D48.1, D50.1, D51.1, D51.3, D54.1, D54.2, D56.2, D61, D63.4, D64, D67.1, D68, D71.1, D74, D80.1 | 31 (49.2) | 21 (33.3) | | 38 (29.9) | 23 (18.1) | |
|  | Caregiving/examination activities | | D2.1, D25, D47.1, D54.1, D58.3, D64, D66.1, D71.1, D79.2, D79.3 |  | 10 (15.9) | |  | 10 (7.9) | |
|  | In a hospital | | D2.1, D2.3, D8, D24, D31.1, D31.2, D37, D42, D43.1, D44.2, D44.3, D47.3, D48.1, D50.1, D50.2, D54.1, D54.2, D57, D58.3, D66.1, D67.1, D68, D72.1, D74, D79.1 |  | 20 (31.7) | |  | 25 (19.7) | |
| Location  n (%) | In a hospital | | D2.1, D2.3, D8, D24, D31.1, D31.2, D37, D42, D43.1, D44.2, D44.3, D47.3, D48.1, D50.1, D50.2, D54.1, D54.2, D57, D58.3, D66.1, D67.1, D68, D72.1, D74, D79.1 | 36 (57.1) | 20 (31.7) | | 48 (37.8) | 25 (19.7) | |
|  | At home | | D6.1, D16, D32.2, D33.1, D47.2, D61, D69.5, D71.1 |  | 8 (12.7) | |  | 8 (6.3) | |
|  | Strange/unknown house | | D11.1, D47.1, D50.1, D51.1, D58.1, D58.2, D63.1 |  | 6 (9.5) | |  | 7 (5.5) | |
|  | Foreign country | | D2.3, D13, D24, D27.1, D35.2, D40.1, D49.1, D63.3, D64, D69.2, D69.3 |  | 10 (15.9) | |  | 11 (8.7) | |
| Employment  n (%) | Participants’ own job | Current | D4.1 | 5 (7.9) | 4 (6.3) | 1 (1.6) | 5 (3.9) | 4 (3.1) | 1 (0.8) |
|  |  | Previous | D9, D49.3, D53.1 |  |  | 3 (4.8) |  |  | 3 (2.4) |
|  | New/other job | | D2.3 |  | 1 (1.6) | |  | 1 (0.8) | |
| Recreational activities  n (%) | Festivities/partying | | D2.3, D67.1, D69.1, D70.1, D80.3 | 10 (15.9) | 5 (7.9) | | 12 (9.4) | 5 (3.9) | |
|  | Outing | | D52.1, D69.2, D69.4 |  | 2 (3.2) | |  | 3 (2.5) | |
|  | Television/theater show | Participation | D68, D74, D79.1 |  | 4 (6.3) | 3 (4.8) |  | 4 (3.1) | 3 (2.4) |
|  |  | Observation | D41 |  |  | 1 (1.6) |  |  | 1 (0.8) |
| Creatures/unreal persons  n (%) | Fantasy figures | Humanlike | D30, D47.1, D47.2, D47.4, 51.1, D51.3 | 13 (20.6) | 8 (12.7) | 3 (4.8) | 18 (14.2) | 12 (9.4) | 6 (4.7) |
|  |  | Monster | D4.2, D15.1, D15.2 |  |  | 2 (3.2) |  |  | 3 (2.4) |
|  |  | Other | D1.1, D56.1, D67.2 |  |  | 3 (4.8) |  |  | 3 (2.4) |
|  | Animals | | D1.2, D13, D37, D54.1, D56.1, D67.2, D69.4, D72.2 |  | 8 (12.7) | |  | 8 (6.3) | |
| Position  n (%) | Lying down | | D24, D25, D31.1, D37, D44.2, D47.1, D47.2, D47.3, D47.4, D49.2, D50.1, D50.2, D51.1, D58.3, D61, D67.2, D68, D70.2, D74, D80.1 | 23 (36.5) | 16 (25.4) | | 28 (22.0) | 20 (15.7) | |
|  | Other specifically mentioned position | | D2.1, D17.1, D19, D21.3, 47.5, D53.1, D64, D66.3 |  | 8 (12.7) | |  | 8 (6.3) | |
| Feeling physically unwell  n (%) | Sick/pain | | D35.2, D61, D74 | 8 (12.7) | 3 (4.8) | | 9 (7.1) | 3 (2.4) | |
|  | Feeling suffocated | | D54.1, D66.1, D66.2, D74 |  | 3 (4.8) | |  | 4 (3.1) | |
|  | Cold | | D27.2, D58.2 |  | 2 (3.2) | |  | 2 (1.6) | |
|  | Tired | | D32.2 |  | 1 (1.6) | |  | 1 (0.8) | |
| Ambient noises  n (%) | People talking | To participant | D10, D27.2, D31.2, D39.2, D41, D61, D64 | 26 (41.3) | 15 (23.8) | 7 (11.1) | 27 (21.3) | 15 (11.8) | 7 (5.5) |
|  |  | Around participant | D2.1, D14.1, D25, D32.1, D37, D54.1, D56.2, D80.1 |  |  | 8 (12.7) |  |  | 8 (6.3) |
|  | Music | | D28, D35.1, D47.2, D72.1 |  | 4 (6.3) | |  | 4 (3.1) | |
|  | Loud noise | | D43.2, D53.1, D74, D79.2 |  | 4 (6.3) | |  | 4 (3.1) | |
|  | Other | | D49.1, D55, D69.3, D72.2 |  | 4 (6.3) | |  | 4 (3.1) | |
| Emotional support  n (%) | Being checked on/being seen | | D1.1, D43.1, D49.3, D79.1 | 7 (11.1) | 4 (6.3) | | 7 (5.5) | 4 (3.1) | |
|  | Being reassured | | D43.1, D44.1, D51.1 |  | 3 (4.8) | |  | 3 (2.4) | |
|  | Being protected | | D14.2 |  | 1 (1.6) | |  | 1 (0.8) | |
